# Editorial: Autologous and Allogeneic Stem Cell Transplant in Cancer Therapy

**DOI:** 10.3390/cancers15051354

**Published:** 2023-02-21

**Authors:** Nidhi Sharma, Yvonne A. Efebera

**Affiliations:** 1 Division of Hematology, The Ohio State University, Columbus, OH 43210, USA; 2 Department of Hematology, Blood and Marrow Transplant, OhioHealth, Columbus, OH 43214, USA

Over the last 10 to 20 years, there have been significant improvements in the fields of both autologous and allogenic transplantation. The upper age limit for hematopoietic stem cell transplantation continues to increase for both autologous SCT (ASCT) and allogeneic HCT (allo-HCT). The improvement in supportive management and development of novel agents has led to increases in survival outcomes in patients undergoing ASCT for multiple myeloma and lymphoma. Advances in conditioning regimens, the expanding use of alternative donor stem cell sources such as haploidentical stem cells and cord blood, and the use of modern T-cell depletion strategies such as post-transplant cyclophosphamide have led to better survival outcomes and a reduced incidence of graft versus host disease in patients undergoing allo-HCT.

This special issue, which comprises 12 papers, addresses various aspects outlining the roles of ASCT and allo-HCT in the treatment of hematological malignancies, new advancements pertaining to their use and the challenges they pose when deciding on the optimal practice.

The paper by Jiang et al. was a retrospective analysis on patients who underwent allo-HCT to better understand how survival has changed over the years. The authors divided the patients into groups based on the year of transplant. The data showed that both progression free and overall survival increased over the years. Five-year graft-versus-host disease/relapse-free survival (GFRS) also increased from 6% to 14% in the latter years. The authors attributed this change to advances in supportive care and treatments focused on the mitigation of graft versus host disease (GVHD) and relapse [1].

For allogenic transplantation, tacrolimus, a calcineurin inhibitor that prevents T-cell activation, is often used for GVHD prophylaxis. However, there is variability in the serum concentrations of tacrolimus (TAC), and little is known on the impact of early TAC levels on acute GVHD (aGVHD). The retrospective study (2002–2016) by the same group studied the effect of early post-transplantation tacrolimus concentration on the risk of acute GVHD. It was reported that achieving ≥10 ng/mL during the first week of allo-HCT may mitigate the risk of aGVHD. However, levels of >11 ng/mL beyond the first week may be associated with suppressed graft versus tumor effect and higher relapse [2].

Graft versus host disease is a serious but common complication associated with allo-SCT as a result of donor T-cell mediated stem cells that attack immunocompromised host tissues. The treatment of aGVHD involves a high dose of corticosteroids, which suppress the immune system and put patients at an even greater risk of infectious complications [3], and potential viral reactivation complications. Despite this increased risk, there are little published data focused on the efficacy of cytomegalovirus (CMV) prophylaxis for patients who develop aGVHD. Wolfe et al. [4] reported on the efficacy of letermovir to prevent clinically significant CMV infection (CS-CMVi) among allo-HCT patients who developed aGVHD in a single center retrospective study. Among aGVHD patients, letermovir prophylaxis decreased CS-CMVi in patients with aGVHD (*p* < 0.001), reduced non-relapsed mortality (*p* = 0.04) and improved overall survival (*p* = 0.04), suggesting that letermovir prophylaxis improves outcomes by preventing CS-CMVi in patients with aGVHD. Dybko et al. aimed to identify predictive and risk factors associated with the increased occurrence of the BK virus-related hemorrhagic cystitis (HC) following hematopoietic stem cell transplantation (HCT). A significant correlation was observed between HC incidences after HCT, BK viremia and viruria, and aGVHD occurrence. Furthermore, the level of BK virus DNA in serum at day +21 (>0.75 × 10^3^) significantly impacted the patients’ survival time [5].

Allo-HCT therapeutic efficacy is mainly dependent on immune alloreactivity mediated by donor lymphocytes infused with the stem cell graft, the so-called graft versus leukemia (GVL) effect. Disease recurrence is the major obstacle to the success of allo-HCT, as patients with high-risk acute leukemia and MDS have a high risk of relapse after allo-SCT [6,7]. Prophylactic donor lymphocyte infusion (DLI) has been shown to reduce the relapse rate but at the cost of increased GVHD [8,9]. Tsirigotis et al. tested the safety and efficacy of a novel method of prophylactic DLI based on prolonged repetitive administration of low lymphocyte doses. When extended for up to 3 years, low-dose pro-DLI administered every two months is safe and effective in reducing relapse rate in patients with high-risk acute leukemia (AL). The data showed that repeated and prolonged DLI administration resulted in relapse prevention, perhaps by inducing a long-lasting anti-leukemic effect [10]. Gutierrez et al., in a retrospective analysis, showed that it may be a curative option in relapsed/refractory (R/R) mantle cell lymphoma with a low cumulative incidence of relapse (CIR). They showed that it might be a better option for fit patients, using human leukocyte antigen (HLA)-identical (related or unrelated) or haploidentical related donors and without previous transplantation. In another study, Tsai et al. compared the outcomes of post cyclophosphamide with or without anti-thymoglobulin and GCSF-primed bone marrow plus peripheral blood stem cells (GIAC). They showed that the mGIAC approach may be a preferential choice for patients with low/intermediate-risk diseases in the view of non-relapse mortality (NRM), CIR, or overall survival (OS) [11].

For multiple myeloma (MM), ASCT is more commonly used compared to allo-HCT. In this issue, three papers were published (two original article and one review). Jordan et al. performed a retrospective survival analysis on newly diagnosed MM (NDMM) patients receiving ASCT from 1992 to 2016 [12]. Patients were split into five groups based on historic changes in novel agents for the treatment of MM. Across the years, there was a statistically significant improvement in both progression-free-survival (PFS) and OS, which was primarily attributed to the inclusion of novel therapies and post-transplantation maintenance. Importantly, significant survival improvements were seen in patients ≤65 years and >65 years old. Baertsch et al. reported on the outcome after salvage high-dose chemotherapy (HDCT)/ASCT following re-induction treatment with carfilzomib/lenalidomide/dexamethasone (KRD). The authors conducted a retrospective analysis of patients that had previously undergone frontline HDCT/ASCT and reported that KRD followed by salvage ASCT were associated with favorable PFS and the response was enhanced by maintenance treatment. Deep remissions achieved with KRD followed by salvage autologous transplantation were associated with favorable PFS and were enhanced by maintenance treatment [13].

In the past years, the therapeutic approaches for patients diagnosed with MM and their respective prognoses have decisively changed with the development of highly efficient new anti-myeloma drugs, such as protosome inhibitors (PI), immunemodulatory drugs (IMID), monoclonal antibodies and CAR-T cells. The review by Greil et al. addresses the role of allo-HCT in MM. The authors discuss data showing that a decreased risk of disease progression may outweigh this treatment-related toxicity for young, fit patients in high-risk constellations with otherwise often poor long-term prognosis. Salvage allo-HCT is recommended, preferentially within clinical trials, for patients with early relapse after first-line therapy including ASCT, and in high risk (HR) constellations according to cytogenetics and stage [14].

Severe acute or chronic GVHD, systemic signs of inflammation and endothelial dysfunction with fluid overload are relatively common posttransplant conditions that are associated with an adverse prognosis and decreased survival due to increased NRM in allotransplant recipients. Previously, it has been shown that there is an association between systemic metabolic profiles and the risk of GVHD, and fluid retention post allo-HCT [15,16,17].

Hatfield et al. studied the potential associations between lipidomic profiles and pretransplant inflammation, early fluid overload and later development of aGVHD. Ninety-two consecutive patients with acute myeloid leukemia (AML) or high-risk myelodysplastic syndrome (MDS) were included in the analysis. The results showed that the pretransplant lipidomic profiles differed significantly when comparing patients with and without the risk factors: (i) pretransplant inflammation, (ii) early fluid overload, and (iii) patients with and without later steroid-requiring aGVHD [18].

The pediatric population treated with allo-HCT most frequently develop endocrine complications. The treatments given, consequently, lead to impaired fitness, including exercise-induced shortness of breath, fatigue and reduced participation in physical activity [19,20]. These symptoms may indicate frailty [21]. Suominen et al. evaluated the physical fitness and prevalence of frailty in male long-term survivors of pediatric allo-transplant. Low muscle strength and a high incidence of frailty were observed in survivors of pediatric allo-HCT. There is a predominant risk of cardiovascular and metabolic diseases in the long-term [22].

We hope that this Special Issue has responded to the clinical demand for up-to-date and in-depth information about the optimal management of patients undergoing hematopoietic stem cell transplantation for hematological malignancies.

## References

[1] Jiang J., Sigmund A.M., Zhao Q., Elder P., Benson D.M., Vasu S., Jaglowski S., Mims A., Choe H., Larkin K. (2022). Longitudinal Survival Outcomes in Allogeneic Stem Cell Transplantation: An Institutional Experience. Cancers.

[2] Sharma N., Zhao Q., Ni B., Elder P., Puto M., Benson D.M., Rosko A., Chaudhry M., Devarakonda S., Bumma N. (2021). Effect of Early Post-Transplantation Tacrolimus Concentration on the Risk of Acute Graft-Versus-Host Disease in Allogenic Stem Cell Transplantation. Cancers.

[3] Broers A.E., van Der Holt R., van Esser J.W., Gratama J.W., Henzen-Logmans S., Kuenen-Boumeester V., Löwenberg B., Cornelissen J.J. (2000). Increased transplant-related morbidity and mortality in CMV-seropositive patients despite highly effective prevention of CMV disease after allogeneic T-cell-depleted stem cell transplantation. Blood.

[4] Wolfe D., Zhao Q., Siegel E., Puto M., Murphy D., Roddy J., Efebera Y., Tossey J. (2021). Letermovir Prophylaxis and Cytomegalovirus Reactivation in Adult Hematopoietic Cell Transplant Recipients with and without Acute Graft Versus Host Disease. Cancers.

[5] Dybko J., Piekarska A., Agrawal S., Makuch S., Urbaniak-Kujda D., Biernat M., Rybka B., Dutka M., Sadowska-Klasa A., Giebel S. (2022). BKV Related Hemorrhagic Cystitis-An Insight into Risk Factors and Later Complications-An Analysis on Behalf of Polish Adult Leukemia Group. Cancers.

[6] Shouval R., Fein J.A., Labopin M., Cho C., Bazarbachi A., Baron F., Bug G., Ciceri F., Corbacioglu S., Galimard J.E. (2021). Development and validation of a disease risk stratification system for patients with haematological malignancies: A retrospective cohort study of the European Society for Blood and Marrow Transplantation registry. Lancet Haematol..

[7] Bazarbachi A., Schmid C., Labopin M., Beelen D., Wolfgang Blau I., Potter V., Niittyvuopio R., Socié G., Blaise D., Sanz J. (2020). Evaluation of Trends and Prognosis Over Time in Patients with AML Relapsing After Allogeneic Hematopoietic Cell Transplant Reveals Improved Survival for Young Patients in Recent Years. Clin. Cancer Res..

[8] Schmid C., Labopin M., Schaap N., Veelken H., Schleuning M., Stadler M., Finke J., Hurst E., Baron F., Ringden O. (2019). Prophylactic donor lymphocyte infusion after allogeneic stem cell transplantation in acute leukaemia—A matched pair analysis by the Acute Leukaemia Working Party of EBMT. Br. J. Haematol..

[9] Scarisbrick J.J., Dignan F.L., Tulpule S., Gupta E.D., Kolade S., Shaw B., Evison F., Shah G., Tholouli E., Mufti G. (2015). A multicentre UK study of GVHD following DLI: Rates of GVHD are high but mortality from GVHD is infrequent. Bone Marrow Transpl..

[10] Tsirigotis P., Gkirkas K., Kitsiou V., Chondropoulos S., Athanassiades T., Thomopoulos T., Tsirogianni A., Stamouli M., Karagiannidi A., Siafakas N. (2021). Repetitively Administered Low-Dose Donor Lymphocyte Infusion for Prevention of Relapse after Allogeneic Stem Cell Transplantation in Patients with High-Risk Acute Leukemia. Cancers.

[11] Gutierrez A., Bento L., Novelli S., Martin A., Gutierrez G., Queralt Salas M., Bastos-Oreiro M., Perez A., Hernani R., Cruz Viguria M. (2022). Allogeneic Stem Cell Transplantation in Mantle Cell Lymphoma; Insights into Its Potential Role in the Era of New Immunotherapeutic and Targeted Therapies: The GETH/GELTAMO Experience. Cancers.

[12] Nunnelee J., Cottini F., Zhao Q., Faisal M.S., Elder P., Rosko A., Bumma N., Khan A., Devarakonda S., Benson D.M. (2022). Improvement in Post-Autologous Stem Cell Transplant Survival of Multiple Myeloma Patients: A Long-Term Institutional Experience. Cancers.

[13] Baertsch M.A., Fougereau M., Hielscher T., Sauer S., Breitkreutz I., Jordan K., Müller-Tidow C., Goldschmidt H., Raab M.S., Hillengass J. (2021). Carfilzomib, Lenalidomide, and Dexamethasone Followed by Salvage Autologous Stem Cell Transplant with or without Maintenance for Relapsed or Refractory Multiple Myeloma. Cancers.

[14] Greil C., Engelhardt M., Finke J., Wäsch R. (2021). Allogeneic Stem Cell Transplantation in Multiple Myeloma. Cancers.

[15] Lindås R., Tvedt T.H., Hatfield K.J., Reikvam H., Bruserud O. (2014). Preconditioning serum levels of endothelial cell-derived molecules and the risk of posttransplant complications in patients treated with allogeneic stem cell transplantation. J. Transpl..

[16] Reikvam H., Grønningsæter I.S., Mosevoll K.A., Lindås R., Hatfield K., Bruserud Ø. (2017). Patients with Treatment-Requiring Chronic Graft versus Host Disease after Allogeneic Stem Cell Transplantation Have Altered Metabolic Profiles due to the Disease and Immunosuppressive Therapy: Potential Implication for Biomarkers. Front. Immunol..

[17] Reikvam H., Grønningsæter I.S., Ahmed A.B., Hatfield K., Bruserud Ø. (2015). Metabolic Serum Profiles for Patients Receiving Allogeneic Stem Cell Transplantation: The Pretransplant Profile Differs for Patients with and without Posttransplant Capillary Leak Syndrome. Dis. Markers.

[18] Hatfield K.J., Bruserud Ø., Reikvam H. (2022). Pretransplant Systemic Lipidomic Profiles in Allogeneic Stem Cell Transplant Recipients. Cancers.

[19] Mertens A.C., Yasui Y., Liu Y., Stovall M., Hutchinson R., Ginsberg J., Sklar C., Robison L.L. (2002). Pulmonary complications in survivors of childhood and adolescent cancer. A report from the Childhood Cancer Survivor Study. Cancer.

[20] Fridh M.K., Simonsen C., Schmidt-Andersen P., Nissen A.A., Christensen J.F., Larsen A., Mackey A.L., Larsen H.B., Müller K. (2021). Cardiorespiratory fitness and physical performance after childhood hematopoietic stem cell transplantation: A systematic review and meta-analysis. Bone Marrow Transpl..

[21] Ness K.K., Krull K.R., Jones K.E., Mulrooney D.A., Armstrong G.T., Green D.M., Chemaitilly W., Smith W.A., Wilson C.L., Sklar C.A. (2013). Physiologic frailty as a sign of accelerated aging among adult survivors of childhood cancer: A report from the St Jude Lifetime cohort study. J. Clin. Oncol..

[22] Suominen A., Haavisto A., Mathiesen S., Mejdahl Nielsen M., Lähteenmäki P.M., Sørensen K., Ifversen M., Mølgaard C., Juul A., Müller K. (2022). Physical Fitness and Frailty in Males after Allogeneic Hematopoietic Stem Cell Transplantation in Childhood: A Long-Term Follow-Up Study. Cancers.

